# The oxidoreductase p66Shc acts as tumor suppressor in BRAFV600E‐transformed cells

**DOI:** 10.1002/1878-0261.12199

**Published:** 2018-05-05

**Authors:** Tobias Furlan, Sana Khalid, Anh‐Vu Nguyen, Julia Günther, Jakob Troppmair

**Affiliations:** ^1^ Daniel Swarovski Research Laboratory Department of Visceral‐, Transplant‐ and Thoracic Surgery Medical University of Innsbruck (MUI) Austria; ^2^ Present address: Oral Biology Department of Dental Medicine University of Pittsburgh Salk Pavilion Room 422A, 335 Sutherland Drive Pittsburgh PA 15213‐2523 USA

**Keywords:** BRAFV600E, intracellular signaling, mitochondria, p66Shc, ROS, vemurafenib resistance

## Abstract

Metabolic reprogramming, as exemplified by the shift from oxidative phosphorylation to glycolysis, is a common feature of transformed cells. In many tumors, altered metabolism is also reflected in increased reactive oxygen species (ROS) levels, which contribute to proliferation and survival signaling. However, despite high ROS levels, cancer cells can be efficiently killed by further increasing ROS production. We have shown previously that both wild‐type and oncogenic CRAF and BRAF prevent excessive mitochondrial ROS production. Subsequently, it has been demonstrated that raising ROS levels in BRAFV600E‐transformed melanoma cells by inhibiting BRAF or MEK rendered them susceptible to cell death induction. To understand how oncogenic BRAF affects mitochondrial ROS production in melanoma, we studied the mitochondrial ROS‐producing oxidoreductase p66Shc, which is frequently overexpressed in tumors. Using NIH 3T3 BRAFV600E fibroblasts and the melanoma cell lines A375 and M238 carrying the same BRAF mutation, we show that under treatment with the ROS‐inducing agent phenethyl isothiocyanate (PEITC), oncogenic BRAF renders cells refractory to p66ShcS36 phosphorylation, which is essential for p66Shc activation and mitochondrial ROS production. Consistent with this, the activation of JNK1/2, which phosphorylate S36, was blunted, while other mitogen‐activated protein kinases were not affected. Inhibition of JNK1/2 efficiently prevented ROS production, while BRAF and MEK inhibitors increased ROS levels. Vemurafenib‐resistant M238R melanoma cells were impaired in S36 phosphorylation and ROS production following PEITC treatment. Moreover, they failed to increase ROS levels after MEK/BRAF inhibition. Finally, shRNA‐mediated knockdown of p66Shc led to increased growth of BRAFV600E‐transformed NIH 3T3 cells in soft agar assay. Taken together, these data suggest that phosphorylation‐activated p66Shc functions as a tumor suppressor in melanoma cells.

**Abbreviations**
ERK: extracellular signal‐regulated kinase
ETC: electron transport chain
JNK: c‐Jun N‐terminal kinase
MAPKs: mitogen‐activated protein kinases
NOX: NADPH‐dependent oxidase
PEITC: phenethyl isothiocyanate
ROS: reactive oxygen species

## 1 Introduction

One hallmark of the progression toward a malignant state in cancer is a metabolic rewiring, which allows tumor cells to cope with the increased need for molecular biosynthesis while maintaining sufficient energy production (Kroemer and Pouyssegur, 2008). A frequently observed feature is the switch to glycolysis even under aerobic conditions. A major driving force behind this are oncogenes and tumor suppressor genes, which through transcription‐dependent and transcription‐independent mechanisms regulate the activity of key metabolic pathways (Rathmell *et al*., 2003; Ward and Thompson, 2012). Additionally, metabolic enzymes themselves may be prone to mutations (Gottlieb and Tomlinson, 2005; King *et al*., 2006). Despite the decline in oxidative phosphorylation, tumors frequently show increased reactive oxygen species (ROS) levels. Apart from mitochondria, NADPH‐dependent oxidases (NOXs), for example, activated through RAS/RAC‐dependent pathways downstream of oncogenic RAS and mutant receptor tyrosine kinases, contribute to this increase (Yang *et al*., 2016b). High levels of ROS favor tumor progression through their DNA‐damaging activity enhancing mutation frequency, while at lower concentration their function as important intracellular signaling molecules predominates (Birben *et al*., 2012; Martindale and Holbrook, 2002; Son *et al*., 2013). Despite their need for increased ROS levels, tumor cells remain exquisitely sensitive to a further rise in ROS (Trachootham *et al*., 2006). Boosting ROS levels to kill tumor cells is part of the effect of cytostatic and radiation therapy (Galadari *et al*., 2017; Gorrini *et al*., 2013; Trachootham *et al*., 2009). However, lowering intracellular ROS levels may also harm tumors due to decreased proliferation and survival signaling (Kim *et al*., 2016; Liu *et al*., 2016).

Other groups, as well as our own, have shown in the past that mitochondrial ROS levels are subject to control by intracellular signaling pathways, which affect ROS production but also the detoxification of ROS by antioxidant systems (Acin‐Perez *et al*., 2009; Ashraf *et al*., 2014; Bensaad and Vousden, 2007; Churchill and Mochly‐Rosen, 2007; Koziel *et al*., 2013; Kuznetsov *et al*., 2008; Liu *et al*., 2008; Piccoli *et al*., 2006; Ramanathan and Schreiber, 2009; Sucher *et al*., 2009; Zhu and Prives, 2009). One source of mitochondrial ROS, which has been extensively studied in the past, is the oxidoreductase p66Shc, which has been linked to redox stress in many pathological settings and diseases (Galimov, 2010). In contrast to other ROS‐generating systems, p66Shc ablation did not interfere with normal cell survival (Giorgio *et al*., 2005), which was not the case for NOX‐dependent ROS production or the inhibition of the electron transport chain (ETC) (Brand *et al*., 2016; Matsushima *et al*., 2013). Enforced expression of p66Shc was sufficient to inhibit the growth of the breast cancer cell line MCF‐7 in a process involving ROS (Yang *et al*., 2016a). Moreover, p66Shc‐dependent induction of ROS by phenethyl isothiocyanate (PEITC) was required for the killing of prostate cancer cells (Xiao and Singh, 2010). In contrast to most other solid tumors, melanomas are characterized by moderate ROS levels (Lebiedzinska‐Arciszewska *et al*., 2015). Melanomas frequently carry an activating mutation in BRAF, most commonly an exchange of valine (V) to glutamic acid (E) at amino acid position 600 (Wellbrock *et al*., 2004; Zebisch *et al*., 2007). In the past, we have shown that signaling through wild‐type (wt) and oncogenic RAF kinases (BRAF and CRAF) prevents excessive mitochondrial ROS production (Kuznetsov *et al*., 2008), without altering the antioxidant capacity of the cells (Koziel *et al*., 2013). Additional published work confirmed the requirement of ROS in the killing of melanoma cells following inhibition of MEK or mutant BRAF (Bauer *et al*., 2017; Verhaegen *et al*., 2006).

We have recently dissected in detail the regulation of p66Shc activation and have provided evidence for the requirement of PKCβ and the MAPKs JNK1 and JNK2 for the phosphorylation activation of p66Shc (Haller *et al*., 2016; Khalid *et al*., 2016). Understanding the crosstalk between oncogene signaling, ROS production by p66Shc, and transformation may identify novel targets for treating melanoma and possibly help to overcome the development of resistance to mutant BRAF‐specific kinase inhibitors (Luke *et al*., 2017).

## 2 Materials and methods

### 2.1 Cell culture, kinase inhibitors, and plasmid transfection

NIH 3T3 (NIH 3T3 wt), NIH 3T3 BRAFV600E (NIH 3T3 V600E), A375, M238, and M238R cells were cultured in T75 flasks containing 20 mL of Dulbecco's modified Eagle's medium (DMEM; Lonza, Basel, Switzerland) with 10% FBS (PAA Laboratories, Coelbe, Germany), 1× penicillin–streptomycin (5000 U·mL^−1^; Thermo Fisher Scientific, Waltham, MA, USA) at 37 °C in a humidified 5% CO_2_/95% air mixture. A375, M238, and M238R are melanoma cell lines which carry the BRAFV600E mutation. M238R cells have been rendered nonresponsive to PLX4032 treatment (Nazarian *et al*., 2010). For microscopic analyses of cells, an 8‐well Nunc Lab‐Tek chamber slide system was used (Thermo Fisher Scientific). The following small‐molecule inhibitors were applied: MEK1/2, AZD6044 (AZD; Axon Medchem, Groningen, NL, 20 μm); BRAFV600E, PLX4032 (PLX; Selleckchem, Munich, Germany, 20 μm); and JNK1/2, SP600125 (SP; Selleckchem, Munich, Germany, 20 μm). DMSO was used as solvent, and an equal amount of DMSO without inhibitor was added to the controls. For knockdown of p66Shc, the plasmids pRetro‐SUPER p66 shRNA (Marco Giorgio, Department of Experimental Oncology, Institute of Oncology, Milan, Italy) and pRetro‐SUPER scrambled (DSL) were used. Cells were seeded in six‐well plates, and up to 1 μg of plasmid DNA was transfected using Lipofectamine (Thermo Fisher Scientific) according to the manufacturer's instructions.

### 2.2 Protein analysis and immunoblotting

Cells were washed twice with PBS, and the pellet was resuspended in 200 μL of ice‐cold NP‐40 lysis buffer (Haller *et al*., 2016; Khalid *et al*., 2016) with 10 μL·mL^−1^ of Protease Inhibitor Cocktail Set I – Calbiochem (Merck Millipore, Billerica, MA, USA). Lysates were spun down at 13 000 ***g*** for 20 min at 4 °C. The supernatant was transferred to a fresh Eppendorf tube, and the protein concentration was measured (Bio‐Rad DC protein assay kit; Bio‐Rad, Hercules, CA, USA). Samples were adjusted to equal protein concentrations, mixed with 6× Laemmli, and heated at 95 °C for 5 min. Up to 60 μg of total protein was separated on 7.5, 10, or 12.5% gels by SDS/PAGE and subsequently blotted onto nitrocellulose membranes. Immunoblotting was carried out as described previously (Haller *et al*., 2016; Khalid *et al*., 2016). The following antibodies were used: Shc1 (610082; BD Biosciences, San Jose, CA, USA), p66S36 (ab54518; Abcam, Cambridge, UK), α‐tubulin (T5168; Sigma, St. Louis, MO, USA). GAPDH (Ambion AM4300; Thermo Fisher Scientific), ERK1/2 (sc‐94; Santa Cruz Biotechnology, Dallas, TX, USA), pERK1/2 (sc‐16982‐R; Santa Cruz Biotechnology), JNK1/2 (sc‐571; Santa Cruz Biotechnology), pJNK1/2 (9251S; Cell Signaling Technology, Leiden, The Netherlands), p38 (9212; Cell Signaling Technology), and pp38 (9211; Cell Signaling Technology). The vinculin antibody was obtained from Santa Cruz (sc‐25336). Detection was done with a fluorescence‐labelled secondary antibodies (Molecular Probes, Eugene, OR, USA). Detection and quantifications of bands were performed by using Odyssey Infrared Imaging System (LiCor Biosciences, Lincoln, NE, USA).

### 2.3 Cell death analysis

Cells were seeded in six‐well plates and treated with PEITC (Sigma‐Aldrich, St. Louis, MO, USA) as indicated. Cells were washed with 2 mL of PBS (PAA Laboratories), detached with 500 μL of trypsin/EDTA (PAA Laboratories), and transferred to 5‐mL Falcon tubes (Corning, Wiesbaden, Germany) together with the supernatant. After centrifugation at 200 ***g*** for 5 min at room temperature, the pellet was resuspended in 30 μL of staining solution containing annexin V (FITC) (Enzo Life Sciences, Farmingdale, NY, USA) and propidium iodide (Carl Roth GmbH Co KG, Karlsruhe, Germany) as described previously (Khalid *et al*., 2016). After incubation, the cells were mixed with 400 μL of binding buffer and centrifuged at 200 ***g*** for 5 min. The supernatant was decanted, and the cells were resuspended in growth medium on ice. The samples were analyzed immediately.

### 2.4 Reactive oxygen species (ROS) measurements

To measure ROS, 60 000 cells were seeded in 8‐well Nunc Lab‐Tek chambers (Thermo Fisher Scientific). The next day, cells were first stressed as indicated and then stained using MitoTracker Red CM‐H2XRos (Life Technologies, Carlsbad, CA, USA) at a concentration of 0.2 μm diluted in serum‐free DMEM, at 37 °C and 5% CO_2_ for 30 min. Before microscopy, cells were resupplied with DMEM/FBS. Digital images were taken using an Olympus IX70 inverted microscope (numerical aperture 0.8) and an Olympus U‐RFL‐T mercury‐vapor lamp (Olympus, Vienna, Austria). Images were acquired using a Kappa ACC1 camera and Kappa imagebase software (Kappa Optronics, Gleichen, Germany). Gray values were quantified using scion image software for Windows (Scion Corporation, Frederick, MD, USA). For every experimental condition, gray values for 80–100 cells were averaged. Alternatively, 500 000 cells were seeded in six‐well plates and treated with different inhibitors for 1 h. To detach cells, they were washed with 2 mL of PBS and treated with 300 μL of trypsin/EDTA for 3 min in the cell culture incubator. Afterward, cells were resuspended in 5 mL of PBS, transferred to FACS tubes, and centrifuged at 200 ***g*** for 5 min. The pellet was resupplied with 1 mL of complete growth medium and inhibitors in addition to different concentrations of PEITC. After 45 min, 4 mL of PBS was added and cells were centrifuged at 200 ***g*** for 5 min. To stain the cells for ROS, the supernatant was decanted and 2′,7′‐dichlorodihydrofluorescein diacetate (DCFDA) (Life Technologies, USA) was added. DCFDA was first dissolved in 100% ethanol and subsequently diluted in serum‐free medium to a concentration of 10 μm. The cells were incubated for 10 min in the tissue culture incubator in the dark. Before FACS analysis, cells were resupplied with 4 mL of complete growth medium and centrifuged at 200 ***g*** for 5 min, and the pellet was resuspended in 400 μL of complete growth medium. FACS analyses were performed on a BD FACSCalibur (BD Biosciences).

### 2.5 Soft agar assay

A 2% SeaPlaque agarose (Biozym Scientific GmbH, Hessisch Oldendorf, Germany) solution dissolved in PBS was autoclaved and diluted to 0.5% with complete growth medium. Two milliliters of 0.5% agar was cast into six‐well plates and allowed to cool down for 15 min at 4 °C to form the bottom agar. Cells were trypsinized, and between 10 000 and 50 000 cells were diluted in 1 mL of complete growth medium. Subsequently, cells were mixed at a ratio of 1 : 1 with 1 mL of 0.8% agar and carefully layered onto the bottom agar. The six‐well plates were left at room temperature for the agar to solidify before incubation in a standard tissue culture incubator. After 24 h, 1 mL of complete medium was carefully added on top of the agar to prevent cells from drying out. After 2 weeks, pictures were taken and colony size was measured using imagej (https://imagej.net/).

### 2.6 Quantitative real‐time reverse transcriptase polymerase chain reaction

Reverse transcriptase polymerase chain reaction (RT‐PCR) for gene expression analysis was performed with the ABI PRISM 7500 Sequence Detection System (Life Technologies, Darmstadt, Germany) as previously described (Ritschl *et al*., 2016). The primers for p66Shc were purchased as TaqMan Gene Expression Assay (Mm00465940m1; Applied Biosystems, Thermo Fisher Scientific, Foster City, CA, USA).

## 3 Results

### 3.1 Impaired p66ShcS36 phosphorylation and ROS levels in BRAFV600E‐transformed cells

**Figure 1:**
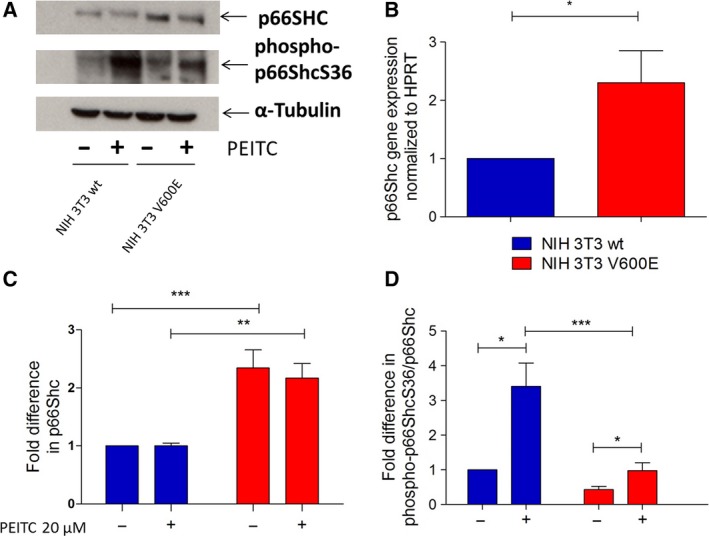
p66Shc status in NIH 3T3 wt and NIH 3T3 V600E‐transformed cells before and after PEITC treatment. A total of 500 000 NIH 3T3 wt and NIH 3T3 V600E cells were seeded in six‐well plates in complete growth medium, and the following day, cells were treated with 20 μm PEITC for 30 min. A representative blot is shown in panel (A). The results of qPCR analysis of p66Shc mRNA expression are summarized in (B). Densitometric analysis of total p66Shc protein relative to α‐tubulin was performed (C). Densitometric analysis of phospho‐p66ShcS36 was performed relative to total p66Shc protein (D). Data are presented as mean value ± standard error of the mean (SEM) of nine independent experiments. *P < 0.1, **P < 0.01, ***P < 0.001.

In a first set of experiments, we tested whether oncogenic BRAFV600E affects the expression or phosphorylation of p66Shc. Immortalized NIH 3T3 fibroblast cells, both parental (NIH 3T3 wt) and those expressing mutant BRAFV600E (NIH 3T3 V600E), were analyzed. While such a cellular model hardly reflects the complexity of cancer cells, it is well suited to study the effects of a single oncogenic mutation on individual aspects of the transformation process. Overexpression of oxidoreductase p66Shc in cancer has been frequently observed (Rajendran *et al*., 2010) and may serve to meet the increased need for higher ROS levels for proliferation and survival signaling. These experiments demonstrated that p66Shc protein expression was increased by approximately 2.5‐fold in BRAFV600E‐transformed cells (Fig. 1A, C), while the expression of the smaller isoforms p52Shc and p46Shc remained unaltered (Fig. S1). A similar increase was also observed for p66Shc mRNA expression (Fig. 1B).

Phosphorylation of p66Shc on serine 36 (S36) is essential for its pro‐oxidant and pro‐apoptotic function (Berniakovich *et al*., 2008; Galimov, 2010; Giorgio *et al*., 2005; Migliaccio *et al*., 1999; Nemoto and Finkel, 2002). Frequently applied chemotherapeutics act via increasing cellular ROS levels, including PEITC used here (Gupta *et al*., 2014; Jutooru *et al*., 2014). Basal p66ShcS36 phosphorylation was lower in BRAFV600E‐transformed cells compared to wt cells (Fig. 1A, D). Moreover, while wt cells responded to PEITC treatment with a pronounced increase in S36 phosphorylation, this effect was negligible in BRAF‐transformed cells (Fig. 1A, D). PEITC had no effect on p66Shc expression levels (Fig. 1A, D).

**Figure 2:**
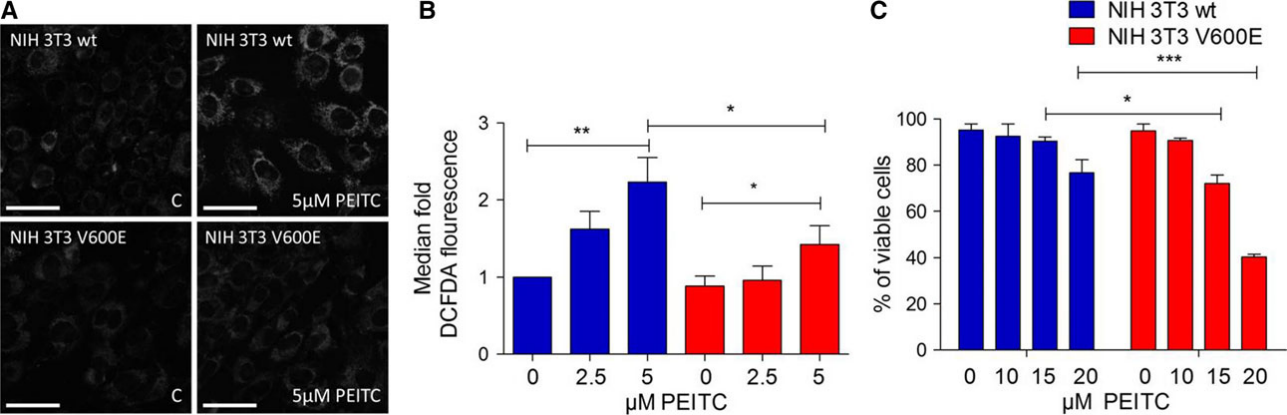
Effect of BRAFV600E on ROS production in PEITC‐treated NIH 3T3 wt and NIH 3T3 BRAFV600E cells. (A) A total of 60 000 cells were seeded in 8‐well Nunc Lab‐Tek chambers and after 20 h treated with PEITC (20 μm) for 30 min and afterward stained with MitoTracker Red CM‐H2XRos. Data are presented as mean value ± standard error of the mean (SEM) of 100 measurements per sample and three independent experiments. Size bar: 50 μm (B) FACS analysis after CM‐H2DCFDA staining of NIH 3T3 wt and NIH 3T3 BRAFV600E cells and PEITC treatment. A total of 500 000 cells were seeded in six‐well plates, and after 30 min of treatment with different concentrations of PEITC, they were stained with CM‐H2DCFDA and subsequently analyzed by FACS. Data are presented as mean value ± standard error of the mean (SEM) of three experiments. (C) A total of 500 000 cells were seeded in six‐well plates and treated overnight with 20 μm PEITC before performing FACS analysis after annexin V staining. Data are presented as mean value ± standard error of the mean (SEM) of three experiments. *P < 0.1, **P < 0.01, ***P < 0.001.

Signaling by oncogenic and wt CRAF and BRAF has been shown in the past to prevent stress‐induced increase in mitochondrial ROS levels (Kuznetsov *et al*., 2008). Therefore, the effect of oncogenic BRAF on PEITC‐induced ROS levels and cell death was studied. A concentration of 5 μm PEITC was sufficient to elicit a significant increase in ROS levels as recorded by confocal imaging following staining with MitoTracker Red CM‐H2XRos (Fig. 2A) or FACS analysis of DCFDA‐stained cells (Fig. 2B) in wt fibroblasts. In contrast, in BRAFV600E‐transformed cells, the increase in ROS production in response to PEITC was significantly lower as compared to their wt counterpart (Fig. 2A, B). We next tested the effect of BRAFV600E mutation on cell survival following PEITC treatment. As described earlier for solid tumor cell lines (Trachootham *et al*., 2006), this fibroblast model of BRAFV600E transformation also showed significantly higher cell death rates than for their normal counterparts (Fig. 2C). Cell death induction by PEITC was prevented in cells pretreated with the antioxidant *N*‐acetyl cysteine, confirming the causative role of ROS (Fig. S2).

### 3.2 MAPK signaling in BRAFV600E‐transformed cells

**Figure 3:**
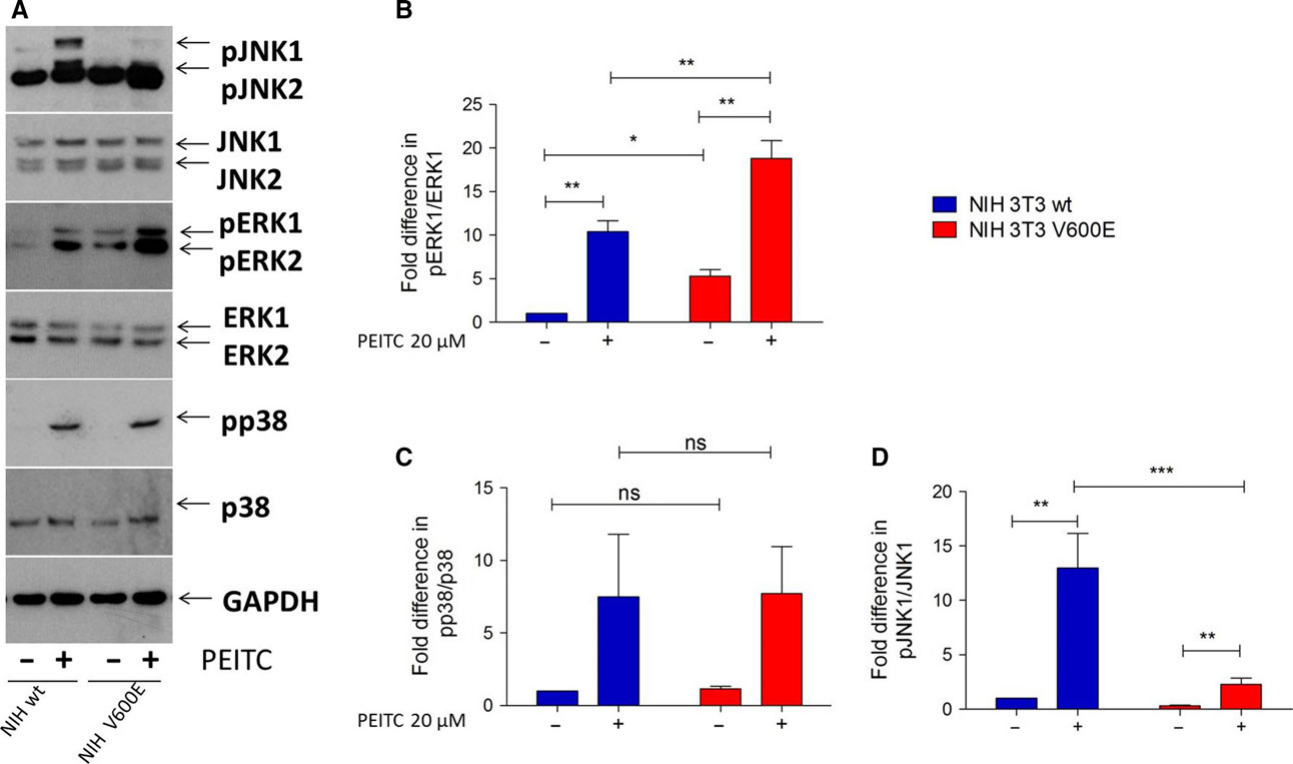
Effect of PEITC treatment on MAPK activity in NIH 3T3 wt and NIH 3T3 BRAFV600E cells. A total of 500 000 cells were seeded in six‐well plates in complete growth medium and treated with 20 μm PEITC for 30 min prior to lysis. Immunoblots were carried out with antibodies specific to phosphorylated ERK1/2 (A, B), p38 (A, C), and JNK1/2 (A, D) and the corresponding antibodies for total protein (A–D). Data are presented as mean value ± standard error of the mean (SEM) of nine independent experiments, except for p38 for which only three experiments were carried out. *P < 0.1, **P < 0.01, ***P < 0.001.

Mitogen‐activated protein kinase (MAPK) signaling is triggered by ROS, but also acts to control ROS levels. A limiting effect on ROS production has been described for signaling through RAF/MEK (Hermann *et al*., 2008; Kuznetsov *et al*., 2008), while activation of the stress kinases p38 and JNK1/2 resulted in increased mitochondrial ROS production (Ashraf *et al*., 2014; Haller *et al*., 2016; Khalid *et al*., 2016). RAF signaling is directly linked to the phosphorylation activation of MEK1/2 and its substrate ERK1/2 (Zebisch *et al*., 2007). Basal ERK1/2 phosphorylation was elevated in BRAFV600E‐transformed cells, but both wt and transformed cell lines responded to PEITC treatment with strongly increased ERK1/2 phosphorylation (Fig. 3A, B). Also p38 activity rose following PEITC treatment of wt and BRAF‐transformed cells to a similar extent (Fig. 3A, C). For JNK1/2, wt cells responded to PEITC treatment with increased JNK1/2 phosphorylation, which was not observed in BRAF‐transformed cells (Fig. 3A, D). Thus, JNK1/2 activation is selectively impaired in BRAF‐transformed cells. To check whether this was restricted to the particular stimulus used here, we also tested sorbitol to activate JNK1/2 and observed the same suppressive effect of BRAFV600E on JNK1/2 activation (Fig. S3).

### 3.3 Effect of MAPK inhibitors on p66ShcS36 phosphorylation and ROS production in wt and NIH 3T3 BRAFV600E‐transformed cells

**Figure 4:**
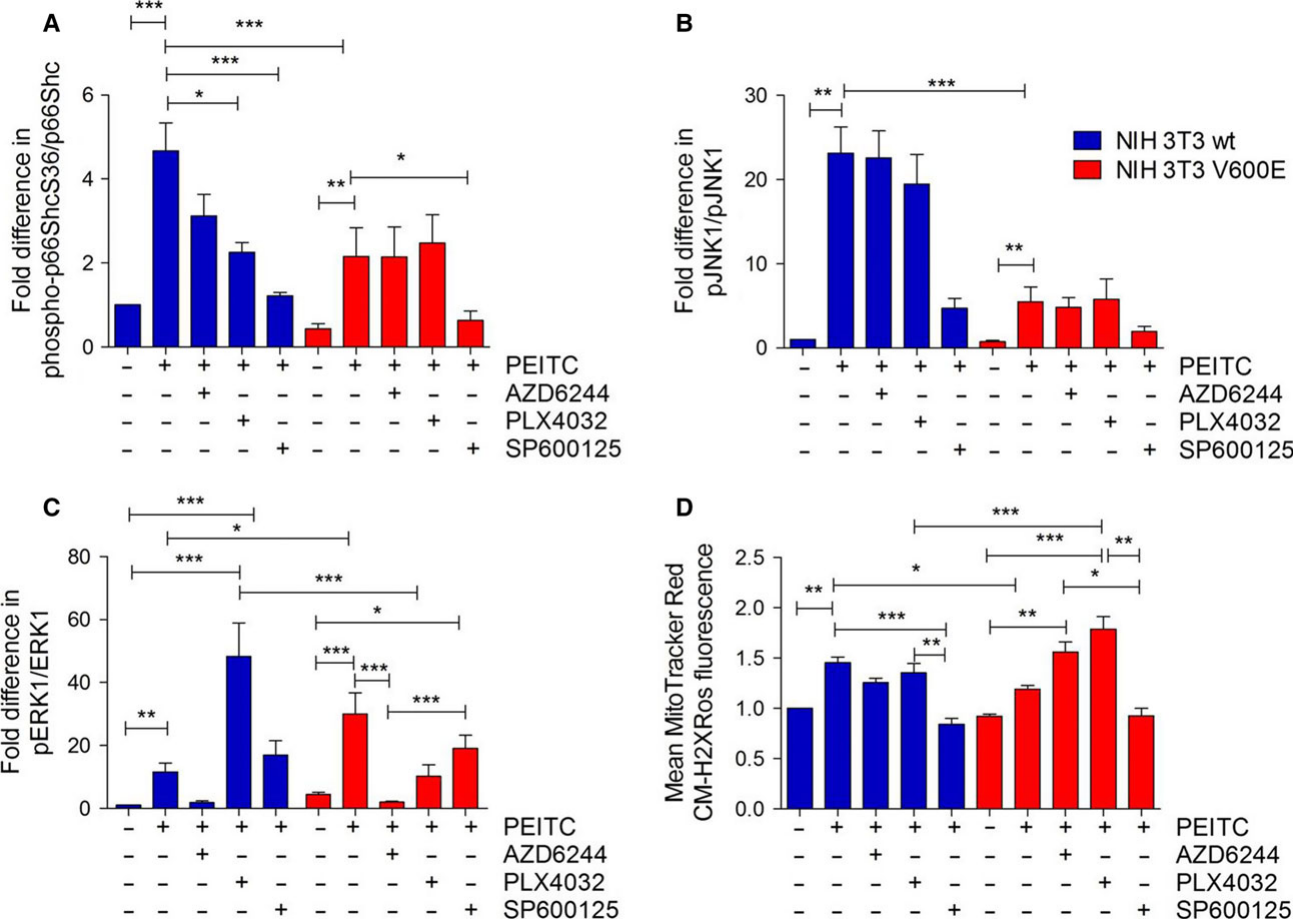
Effect of MAPK inhibition on p66Shc activation and ROS production. A total of 500 000 cells were seeded in six‐well plates in complete growth medium and treated with 20 μm of AZD6244, PLX4032, or SP600125, 1 h prior to PEITC treatment (30 min, 20 μm) prior to lysis. Immunoblots were carried out for the proteins indicated, and phosphorylation of p66Shc (A), JNK1 (B), or ERK1 (C) was quantified densitometrically. Data are presented as mean value ± standard error of the mean (SEM) of nine independent experiments. (D) ROS production was quantified as described in Fig. 2D. Data are presented as mean value ± standard error of the mean (SEM) of 100 measurements per sample and three experiments. *P < 0.1, **P < 0.01, ***P < 0.001.

We next analyzed the effect of inhibitors of MEK1/2 (AZD6244, AZD), BRAFV600E (PLX4032, PLX), and JNK1/2 (SP600125, SP) on p66ShcS36 phosphorylation following PEITC treatment. Among the inhibitors tested, PEITC‐induced S36 phosphorylation in wt cells was partially inhibited by AZD and PLX and almost completely blocked by SP (Fig. 4A). As seen above (Fig. 1B), S36 phosphorylation was lower in transformed cells and unaffected by the inhibitors of MEK1/2 and oncogenic BRAF, while the inhibition of JNK1/2 completely prevented S36 phosphorylation (Fig. 4B). When analyzing JNK1/2 activation in the same samples, no significant effect was observed in the case of BRAF/MEK inhibitors, while SP demonstrated pronounced inhibition (Fig. 4B). This suggests that JNK1/2 activation does not depend on BRAF/MEK signaling. AZD had the expected effect on ERK1/2 phosphorylation, while PLX increased ERK1/2 phosphorylation in wt cells in agreement with published data (Hatzivassiliou *et al*., 2010), whereas decreased ERK phosphorylation was observed in NIH 3T3 BRAFV600E cells (Fig. 4C). Elevated ROS levels were observed in NIH 3T3 BRAFV600E cells following the inhibition of BRAF or MEK (Fig. 4D), while the inhibition of JNK1/2 prevented ROS production (Fig. 4D).

**Figure 5:**
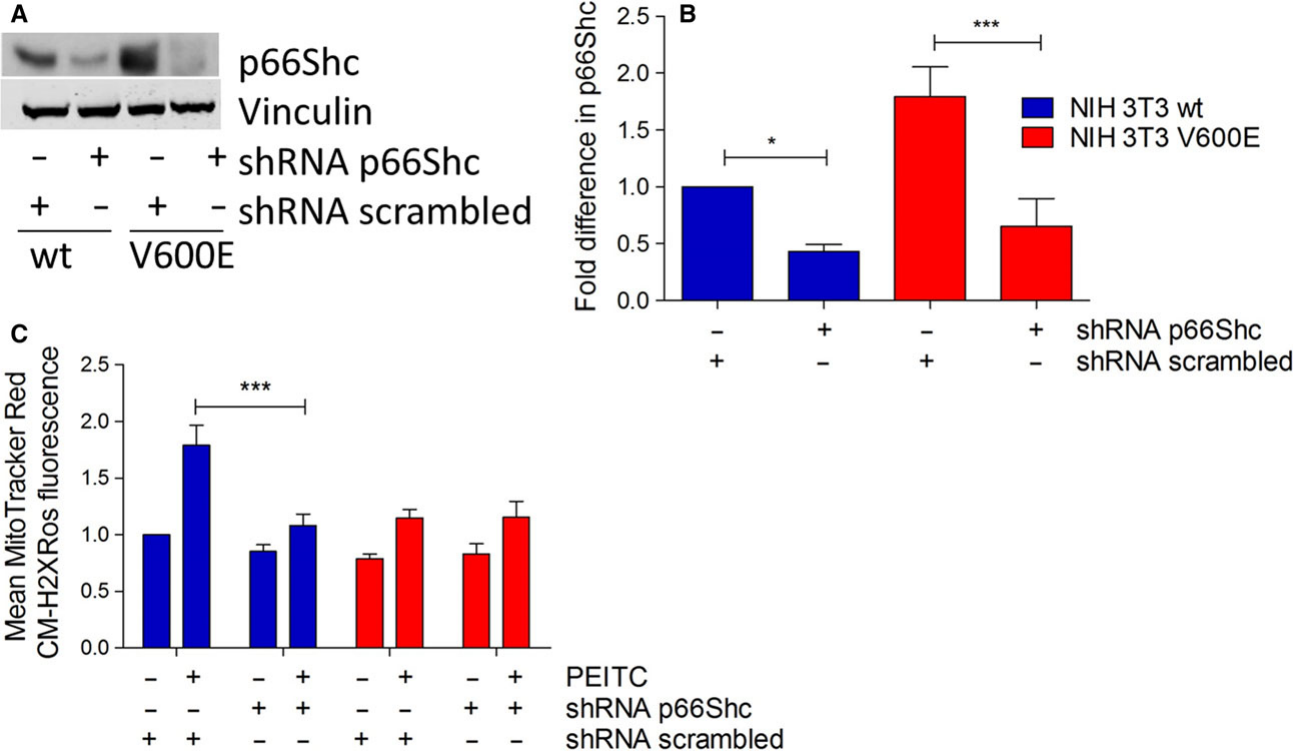
p66Shc is required for PEITC‐induced ROS production. NIH 3T3 wt and NIH 3T3 V600E cells were transfected with plasmids expressing either scrambled or p66Shc‐specific shRNA and selected with puromycin. For immunoblots, cells were seeded in six‐well plates in complete growth medium at a density of 600 000 cells per well and treated with 20 μm PEITC for 30 min prior to lysis. p66Shc expression was analyzed by immunoblotting (A) and normalized to loading control (B). Fluorescence microscopy of ROS levels was performed after 30 min of 5 μm PEITC stress and MitoTracker Red CM‐H2XRos staining. Data are presented as mean value ± standard error of the mean (SEM) of 100 measurements per sample and three experiments (C). *P < 0.1, ***P < 0.001.

To further confirm that p66Shc really is a major contributor to ROS production in the cell model studied, stable shRNA‐mediated knockdown of p66Shc was performed in wt and BRAFV600E‐transformed fibroblasts and confirmed by immunoblotting (Fig. 5A, B). At the p66Shc protein level, a reduction by 50% was achieved in wt and transformed cells. This knockdown completely blocked PEITC‐induced ROS production in wt cells (Fig. 5C). BRAFV600E‐transformed cells again demonstrated significantly lower ROS levels following PEITC treatment, which were not further affected by p66Shc knockdown.

### 3.4 Regulation of ROS levels in BRAFV600E‐mutant melanoma cells

**Figure 6:**
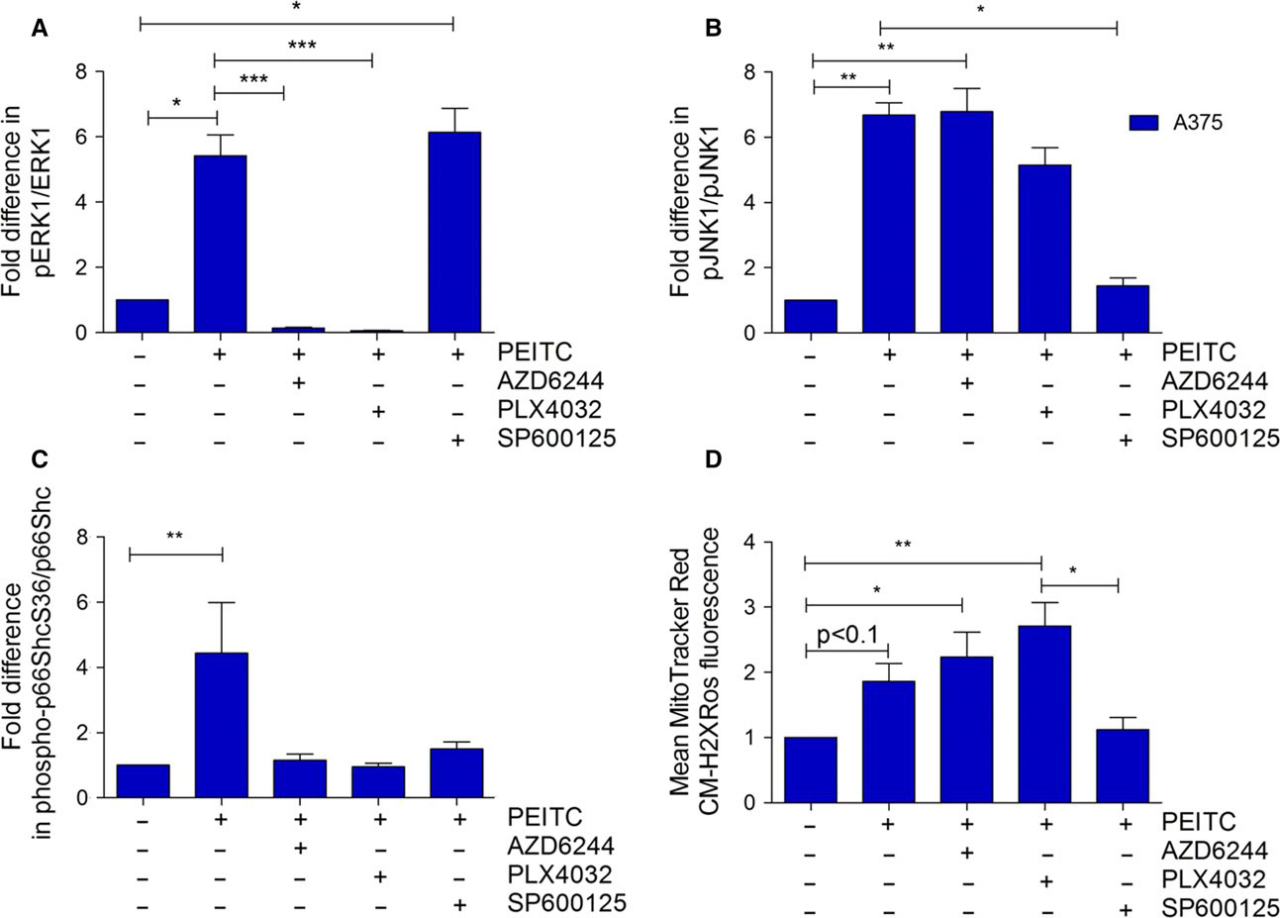
p66Shc and MAPK activity in A375 after combined PEITC and inhibitor treatment. A375 cells were seeded in six‐well plates at a density of 500 000 cells per well and treated with 20 μm of AZD6244, PLX4032, or SP600125, 1 h prior to treatment with 20 μm PEITC for 30 min. Immunoblots were performed using phosphorylation‐specific and total protein antibodies for ERK1 (A), JNK1 (B), and p66Shc (C). Results presented are from five independent experiments. (D) For ROS measurements, cells were seeded in 8‐well chamber slides and treated as described above before staining with 100 μm MitoTracker Red CM‐H2XRos for 10 min. Data are presented as mean value ± standard error of the mean (SEM) of 100 measurements per sample and three experiments. *P < 0.1, **P < 0.01, ***P < 0.001.

To further study signaling interactions, we switched to A375 cells, a melanoma cell line carrying the BRAFV600E mutation. A375 cells responded to PEITC treatment with ERK1 phosphorylation but also with the activation of JNK1 (Fig. 6A, B). Inhibiting BRAFV600E, MEK1/2, or JNK reduced S36 phosphorylation (Fig. 6C), while only SP was efficient in inhibiting ROS production. Again, BRAF and MEK inhibition resulted in increased ROS levels (Fig. 6D).

**Figure 7:**
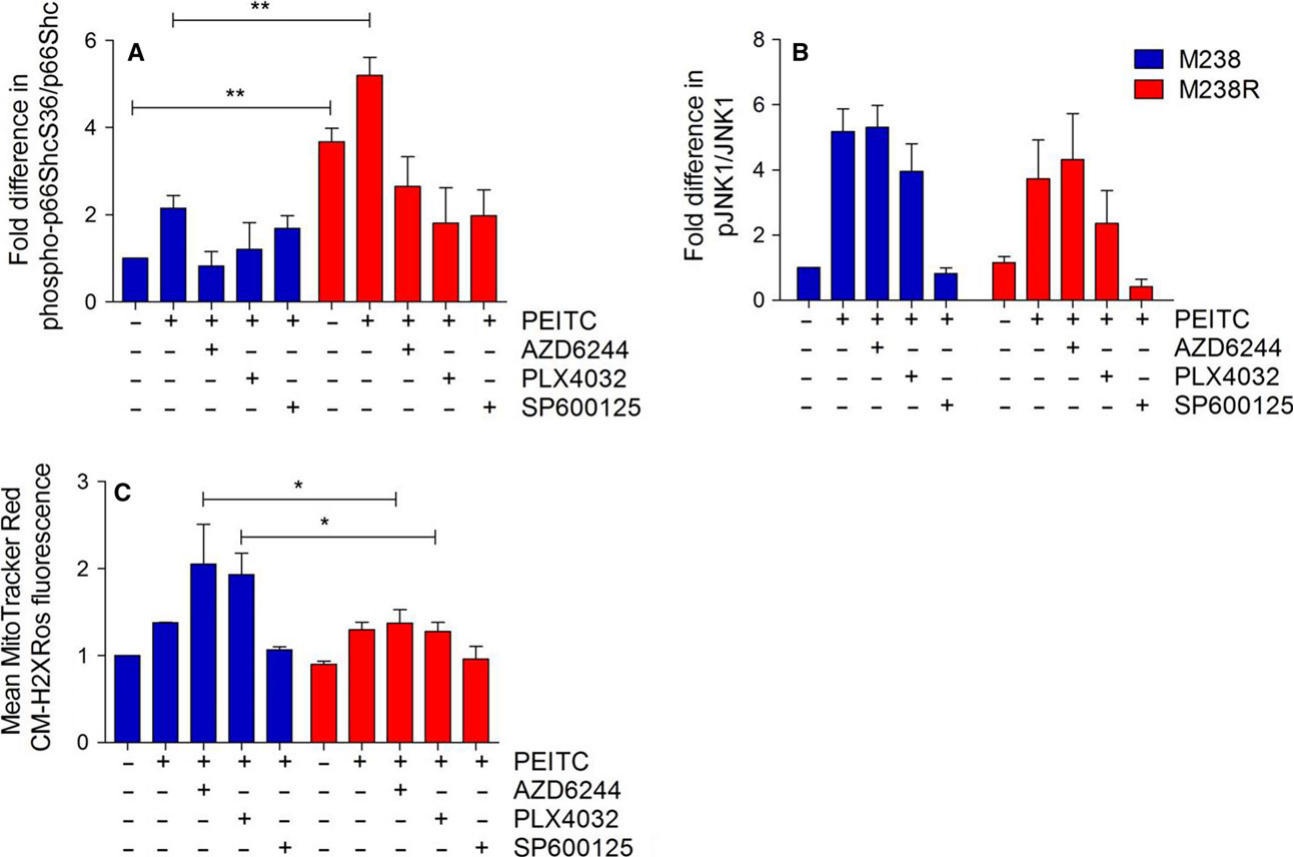
p66Shc and MAPK activity in M238/M238R after combined PEITC and inhibitor treatment. Cells were seeded in six‐well plates at a density of 500 000 cells per well and treated with 20 μm of AZD6244, PLX4032, or SP600125, 1 hour prior to treatment with 20 μm PEITC for 30 min. Immunoblots were performed using phosphorylation‐specific and total protein antibodies for p66Shc (A) and JNK1 (B). (C) For ROS measurements, cells were seeded in 8‐well chamber slides and treated as described above before staining with 100 μm MitoTracker Red CM‐H2XRos for 10 min. Data are presented as mean value ± standard error of the mean (SEM) of 100 measurements per sample and three experiments. *P < 0.1, **P < 0.01.

In a next set of experiments, we analyzed MAPK signaling, p66Shc activation, and ROS production in a pair of cell lines which either respond to vemurafenib treatment (M238) or have become resistant to it (M238R) (Nazarian *et al*., 2010). PEITC‐induced increase in phospho‐p66ShcS36 in parental cells was comparable to what had been observed in BRAF‐transformed fibroblasts (Fig. 7A). Basal S36 phosphorylation was about fourfold higher in M236R cells and only marginally increased after PEITC treatment (Fig. 7A). Application of all inhibitors tested in this study reduced S36 phosphorylation (Fig. 7A), and JNK1 activity was equal in both cell lines and only responded to JNK inhibition (Fig. 7B). Finally, when comparing ROS production, our data showed that vemurafenib‐resistant cells failed to respond to PEITC treatment with a pronounced increase in ROS, not even after BRAF/MEK inhibition (Fig. 7C). Thus, despite JNK activation and S36 phosphorylation, vemurafenib‐resistant melanoma cells failed to respond with ROS production.

### 3.5 Effect of p66Shc knockdown on BRAFV600E transformation

**Figure 8:**
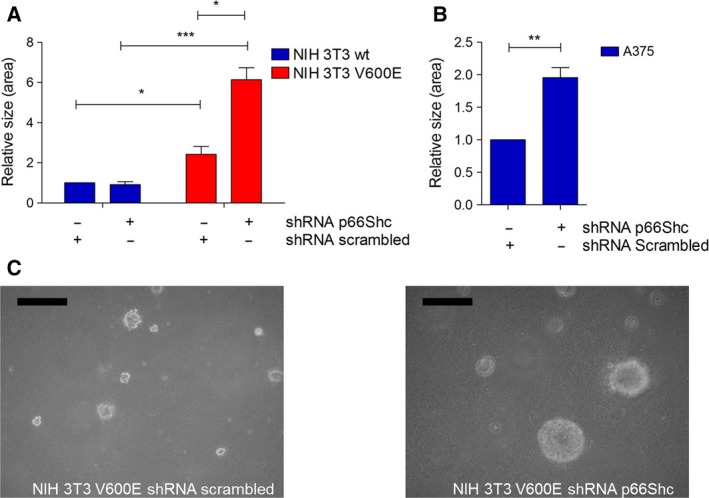
Soft agar growth of NIH 3T3 V600E and A375 cells. Cells were transfected with plasmids expressing shRNA (p66Shc or scrambled) and puromycin resistance. Cells with stable plasmid expression were selected before seeding 10 000 cells in soft agar in six‐well plates in soft agar. After 7 days, the size of the colonies was assessed by measuring the surface area of the colonies using imagej. NIH wt and V600E cells (A) as well as A375 cells (B) were used. NIH 3T3 cells served as control and formed no colonies in soft agar. A representative picture of V600E cells expressing shRNA scrambled (left) or p66Shc (right) is shown in panel (C). Size bar: 500 μm. *P < 0.1, **P < 0.01, ***P < 0.001.

The data presented so far suggest that expression of oncogenic BRAFV600E blunts ROS production by preventing the activation of p66Shc. To further study the contribution of p66Shc to transformation, we performed a stable shRNA‐mediated knockdown in BRAFV600E‐transformed NIH 3T3 and A375 cells and tested the effect on growth in soft agar. As shown in Fig. 8A, B, the knockdown of p66Shc greatly enhanced proliferation of transformed cells, further supporting a tumor‐suppressive effect of p66Shc. Representative pictures are shown in Fig. 8C.

## 4 Discussion

Metabolic rewiring to meet the increased need for biosynthesis is a common feature of transformed cells, which also opens up novel possibilities for therapeutic intervention. Most commonly, these changes affect key metabolic pathways, but also the increased production of ROS may be a direct result of these adaptations (Berger *et al*., 2017; Galluzzi *et al*., 2013; Kroemer and Pouyssegur, 2008; Ratnikov *et al*., 2017). The whole process is driven by oncogenic signaling pathways long implicated in the transformation process, but also metabolic enzymes are possible targets for mutation. Signaling through the small GTPase RAS and upstream oncogenes has been linked to the activation of NOX and thus increased ROS production (Bokoch and Knaus, 2003; Kamata, 2009). Other contributors may be the mitochondria themselves where mutations result in mitochondrial dysfunction causing increased ROS production (Yang *et al*., 2016b). Targeting ROS for therapeutic intervention is faced with the danger of tilting the balance to insufficient or excessive ROS levels also in nontransformed cells, which are both incompatible with cell survival. Knockout studies of NOX and inhibition of ROS production by the ETC of the mitochondria have highlighted the imminent danger of these approaches (Brand *et al*., 2016; Matsushima *et al*., 2013). In the case of p66Shc, the benefit of eliminating p66Shc function for the prevention of oxidative stress‐induced organ damage has been demonstrated for many disease settings, while no negative effects on normal physiological processes were noted (Giorgio *et al*., 2005). Also, no compensation by other ROS‐producing systems was observed under these conditions. Inhibiting p66Shc function thus may be the preferred strategy for the prevention of pathologies associated with excessive ROS production, while activating p66Shc may be an approach to raise ROS levels in tumors in order to cause cell death.

We have shown previously that signaling through wt and activated CRAF and BRAF prevented excessive mitochondrial ROS levels (Kuznetsov *et al*., 2008). Inhibition of mutant BRAF in melanoma caused an increase in ROS production, which facilitated cell killing (Bauer *et al*., 2017; Verhaegen *et al*., 2006). In the work presented here, we provide evidence for a link between the presence of BRAF mutation and the inability of these cells to activate p66Shc. As we observe the same effect in BRAFV600‐transformed fibroblasts and melanoma cells carrying the same mutation, oncogenic BRAF on its own is sufficient to prevent p66Shc activation. With regard to the underlying mechanisms, our results point to a specific defect in the activation of JNK1/2 kinases, which are essential for phosphorylation activation of p66Shc. The reasons for this are still currently unknown. Cross talk between MAPKs signaling pathways has been reported and may involve phosphorylation and dephosphorylation events at different levels of the signaling cascades resulting in various outcomes with regard to activation of suppression of signal flow (Junttila *et al*., 2008; Kang *et al*., 2017; Lopez‐Bergami *et al*., 2007; Meng and Xia, 2011; Shen *et al*., 2003). In our experiments, we observe ROS‐dependent killing of BRAFV600E‐transformed cells despite their impaired ability for ROS production following PEITC stimulation. The increased sensitivity of transformed cells (in this case by the RAF upstream oncogenes KRASV12 and BCR‐ABL) to treatments further increasing ROS levels has been reported before (Trachootham *et al*., 2006). RAF‐transformed cells were not addressed in this study, but we have previously reported that oncogenic RAF kinases will rather prevent excessive ROS production (Kuznetsov *et al*., 2008), as we observe here. Our data thus would suggest that the limited increase in ROS levels, which is observed in transformed cells, is sufficient for cell death induction. The underlying mechanisms remain to be addressed in future experiments.

The inability to activate JNK1/2 in BRAFV600E‐transformed cells is not limited to the specific stimulus tested here, but was also seen when sorbitol was used to activate JNK1/2. In A375 cells, we observed that inhibition of BRAF/MEK also prevented S36 phosphorylation. Phosphorylation of S36 by ERK1/2, downstream of BRAF/MEK, appears possible, as this site conforms to a consensus site for phosphorylation by proline‐directed kinases (Fujii *et al*., 2004) and ERK phosphorylation of S36 has been reported, for example, in glomerular mesangial cells after endothelin‐1 stimulation (Foschi *et al*., 2001). We currently do not know why only JNK1/2 but not BRAF/MEK inhibition prevented ROS production. It appears possible that the increase in ROS levels as a consequence of BRAF/MEK inhibition, through p66Shc‐independent mechanisms, surpasses the effect of p66Shc inhibition on overall ROS levels. The situation may be even more complex in cells which have become resistant to vemurafenib treatment. They show elevated p66Shc S36 phosphorylation but still fail to produce ROS. One possible explanation may be our previous demonstration that S36 phosphorylation by JNK1/2 is insufficient for full p66Shc activation but requires additional phosphorylation by PKCβ (Haller *et al*., 2016).

The notion that the inability to activate p66Shc has tumor‐suppressive function in melanoma cells is further supported by the results of the p66Shc knockdown in melanoma cells, which resulted in enhanced growth of melanoma cells in soft agar. This also suggests that restoring p66Shc activation may hold the key to a more efficient melanoma therapy. Understanding the mechanisms of p66Shc activation by upstream kinases JNK1/2 and PKCβ (Haller *et al*., 2016; Khalid *et al*., 2016), as we have recently investigated, holds the key for inhibition of p66Shc activation as it may be warranted in pathological settings characterized by excessive ROS production. Increasing ROS in cancer to induce cell death by selectively activating specific signaling pathways with cell‐penetrating compounds is technically not feasible and most likely also would not overcome the block in JNK1/2 activation observed in our cellular models. A more feasible approach has been recently suggested by the demonstration that the breast cancer cell line MCF‐7 can be killed by infection with viral p66Shc expression vectors. To circumvent the problem in p66Shc activation due to lacking JNK1/2 activity, a mutant form of p66Shc carrying glutamic acid (E) instead of serine (S) in position 36 could be used. This mutant has been shown to restore ROS production to mouse embryonic fibroblasts deficient in JNK1/2 (Khalid *et al*., 2016). That melanoma cells in general may be protected against an increase in p66Shc‐dependent ROS production is also supported by the demonstration that the protein melanoma inhibitory activity (MIA) antagonizes p66Shc in melanoma (Kasuno *et al*., 2007). Inhibition of MIA by a synthetic peptide both decreased the number of metastases and led to immunosuppression in a murine model of malignant melanoma (Riechers and Bosserhoff, 2014).

## 5 Conclusion

Taken together, these data suggest that melanoma may be intrinsically deficient in mitochondrial ROS production by p66Shc. Overcoming this resistance may hold the key to novel therapies for the treatment of this cancer entity and may also be feasible in tumors resistant to currently used mutant BRAF inhibitors.

## Author contributions

JT designed the study and drafted the final manuscript; TF, SK, and JT planned the experiments; TF, SK, AN, and JG performed the experiments, compiled, and analyzed the data; TF prepared the final figures. All authors read and approved the final manuscript.

## Supporting information


**Fig. S1.** Expression of the p46 and p52 isoforms of Shc is not affected by BRAFV600E.

**Fig. S2.** *N*‐acetyl cysteine (NAC) prevents PEITC‐induced cell death.

**Fig. S3.** BRAFV600E‐transformed cells are impaired in JNK1/2 activation following stimulation with sorbitol.
