# Supplementary figures and images for: The oxidoreductase p66Shc acts as tumor suppressor in BRAFV600E‐transformed cells
Source: Mol Oncol. 2018 May 5;12(6):869–82. doi: 10.1002/1878-0261.12199 (PMC5983121; doi:10.1002/1878-0261.12199)

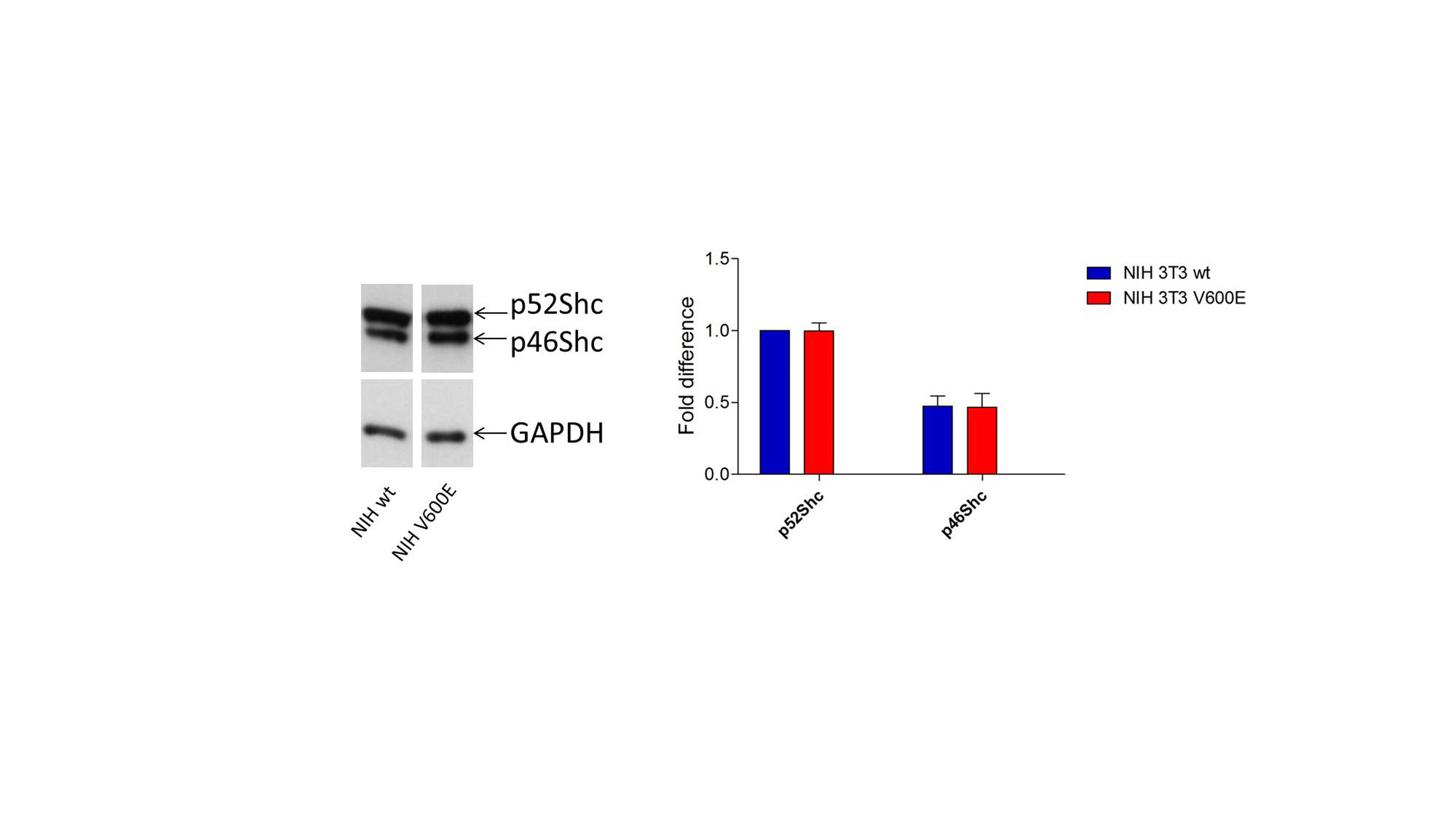

Supplement: Supplementary file 1 — Fig. S1. Expression of the p46 and p52 isoforms of Shc is not affected by BRAFV600E. [file MOL2-12-869-s001.tif]

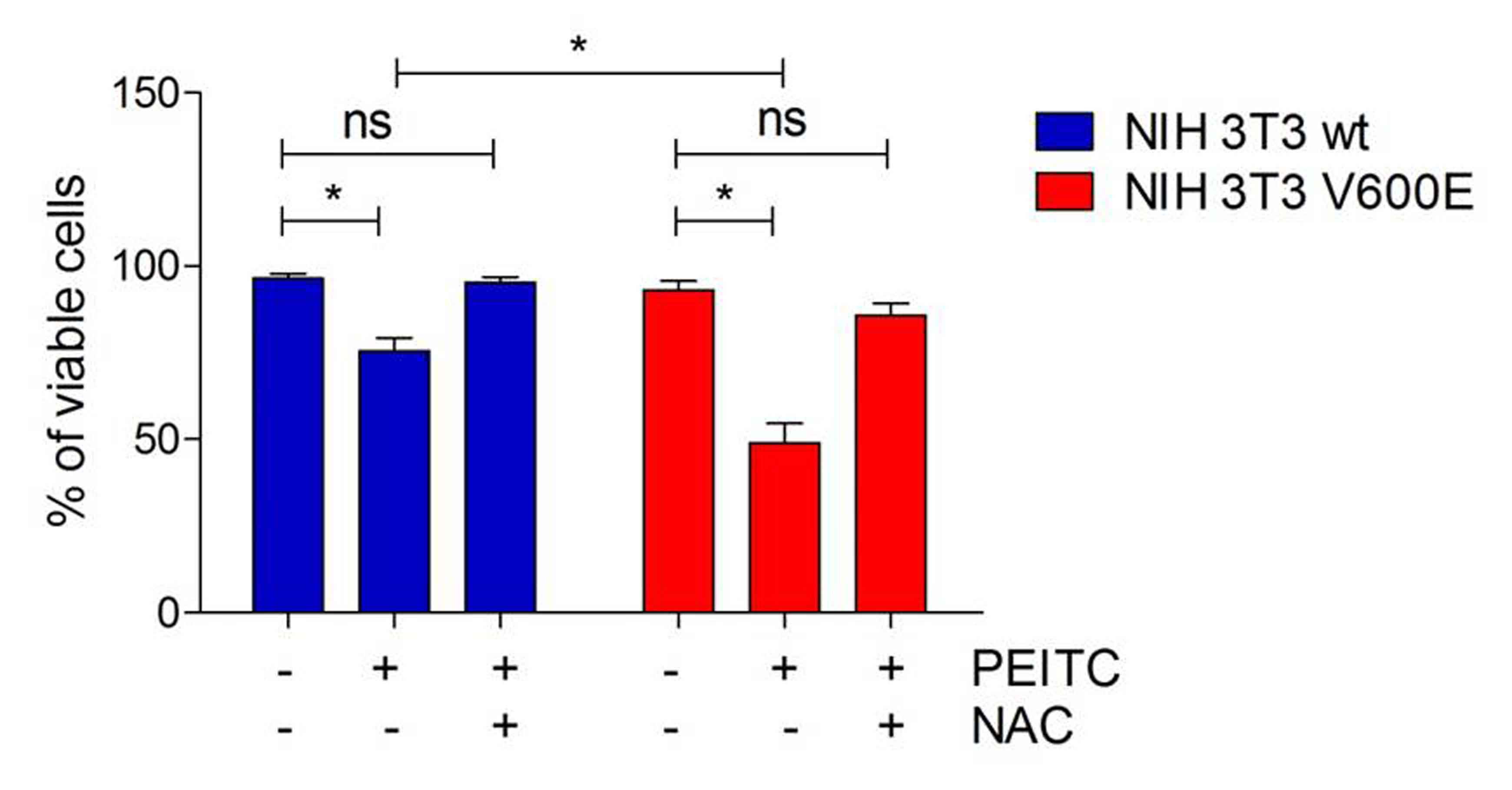

Supplement: Supplementary file 2 — Fig. S2. N‐acetyl cysteine (NAC) prevents PEITC‐induced cell death. [file MOL2-12-869-s002.tif]

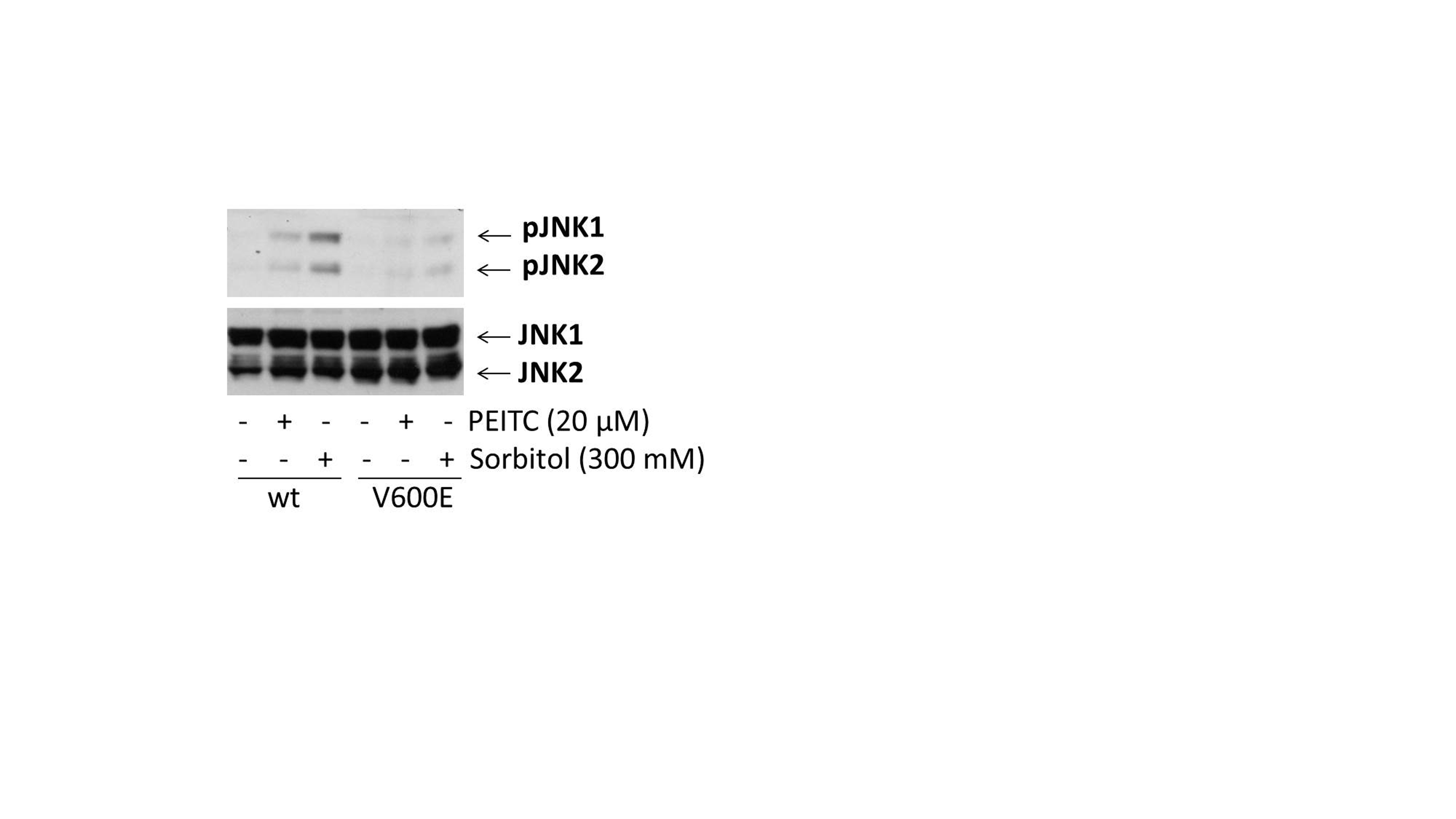

Supplement: Supplementary file 3 — Fig. S3. BRAFV600E‐transformed cells are impaired in JNK1/2 activation following stimulation with sorbitol. [file MOL2-12-869-s003.tif]
